# Professional identity work of nurse practitioners and family physicians in primary care in Quebec and Ontario – a study protocol

**DOI:** 10.1186/s12875-024-02415-3

**Published:** 2024-05-21

**Authors:** Charo Rodríguez, Douglas Archibald, Roland Grad, Katya Loban, Kelley Kilpatrick

**Affiliations:** 1 Department of Family Medicine, School of Medicine, Faculty of Medicine and Health Sciences, McGill University, 5858 Côte-des-Neiges Road, 3rd floor, suite 300, room 328, Montreal, QC H3S 1Z1 Canada; 2 Institute of Health Sciences Education, Faculty of Medicine and Health Sciences, McGill University, Montreal, QC Canada; 3 Department of Family Medicine, Faculty of Medicine, University of Ottawa, Ottawa, ON Canada; 4 Bruyère Research Institute, Ottawa, ON Canada; 5 Research Institute of the McGill University Health Center, Montreal, QC Canada; 6 Ingram School of Nursing, Faculty of Medicine and Health Sciences, McGill University, Montreal, QC Canada

**Keywords:** Professional identity, Family medicine, Nursing, Interdisciplinary primary health care

## Abstract

**Background:**

Solo medical practices in primary healthcare delivery have been abandoned in favor of interdisciplinary teamwork in most Western countries. Dynamics in interdisciplinary teams might however be particularly difficult when two or more autonomous health professionals develop similar roles at the practice level. This is the case of family physicians (FPs) and nurse practitioners (NPs), due to the fact that the latter might accomplish not only the traditional role proper to a nurse, but also several medical activities such as requesting diagnostic exams and prescribing medical treatments. The tensions that this overlap might generate and their implications in regard of the development of professional identities, and consequently of the quality of health care delivered, have been suggested, but rarely examined empirically. The goal of this study is to examine identity work, i.e., the processes of (re)construction of professional identities, of NPs and FPs working together in primary care interdisciplinary teams.

**Methods:**

A longitudinal, interpretive, and comparative multiple (*n* = 2) case study is proposed. Identity work theory in organizations is adopted as theoretical perspective. Cases are urban primary care multidisciplinary teams from two different Canadian provinces: Quebec and Ontario. Participants are NPs, FPs, managers, and patients. Data gathering involves audio-diaries, individual semi-structured and focus group interviews, observations, and archival material. Narrative and metaphor techniques are adopted for analyzing data collected. Within- and cross-case analysis will be performed.

**Discussion:**

For practice, the results of this investigation will: (a) be instrumental for clinicians, primary care managers, and policy decision-makers responsible for the implementation of interdisciplinary teamwork in primary healthcare delivery to improve decision-making processes and primary care team performance over time; (b) inform continuing interdisciplinary professional development educational initiatives that support competency in health professionals’ identity construction in interdisciplinary primary care organizations. For research, the project will contribute to enriching theory about identity construction dynamics in health professions, both in the fields of health services and primary care education research.

## Background

In a broad context in which the boundaries among professions are becoming more and more permeable [15, 22], a better understanding of the ‘blurriness’ in the identity role of primary health care providers, its tensions and implications for the professionals involved, their professional practice, health care delivery, and ultimately patient health outcomes is required. Our overarching goal in this study is to examine active processes of professional identity (re)construction (i.e., identity work) in the context of interdisciplinary primary care teams due to task sharing between family physicians (FPs) and specialized nurse practitioners (NPs). More specifically, the study is guided by the following intertwined research questions: (Q1) What is the meaning that FPs and NPs give to their respective professional identities in primary care? (Q2) What and how do individual, team, organizational, and institutional factors influence their FPs and NPs identity work in interdisciplinary primary care teams?

Professional identity, which is constantly revisited in situated work settings [10], is of paramount importance for organizations because, as underscored by Alvesson et al. [5], it ”can be linked to nearly everything: from mergers, motivation and meaning-making to ethnicity, entrepreneurship and emotions to politics, participation and project teams” (p. 5). Identity research might therefore shed light on individual behavior in organizational settings, understand labor experiences and well-being, unveil, and recognize “the ‘darker’ aspects of organizational life”, and ultimately support managerial practices aimed to enhance organizational performance [5, 7, 32].

In the health care context, the abandonment of solo medical practice in primary care in favor of interdisciplinary teamwork has become an international trend driven by policy reforms in most Western countries [73, 74, 84]. The benefits of this shift have been well documented [74], but also the important professional and organizational challenges that it carries [33]. Interdisciplinary teamwork dynamics might be particularly difficult when two (or more) self-defined autonomous health providers develop similar roles at the practice level. Among others, this is the case of FPs and NPs, as the latter might now perform not only the traditional nursing role, but also medical activities (such as prescribing diagnostic tests and treatments) that have traditionally been under the purview of FPs. Identity tensions among members of interdisciplinary clinical teams have been identified [11, 58, 85], but these processes, and their implications, are still poorly understood. Professional identity tensions might, for instance, disrupt the personal continuity of service provision, when current evidence clearly points out its benefits for better patient health outcomes [8]. Advancing knowledge on professional identity development to better equip FPs and NPs to work together within primary health care teams is therefore of paramount importance, in particular when complex patients and their families are involved. If this ideal way of working together is not well realized, personal continuity of care will decrease. This will likely result in ‘too much health care’ via issues such as over-testing and overdiagnosis [47].

### Literature Review

#### Identity

*Definition*. Identity is a complex construct that has been examined by scholars of different disciplines (e.g., anthropology, sociology, psychology, organizational studies), and from a variety of perspectives (e.g., social identity theory, ethnicity and gender research, critical theory) [62, 82]. Over the last decades, there has been a renewed concern for identity issues, largely justified by broader changes in society (globalization, the expansion of information technology, and greater diversity), the decline of bureaucratic organizational forms, and a special interest in power and meaning [1, 5, 14, 21, 62]. An extensive stream of work in sociology and social psychology has viewed identity as a well-defined, salient, and enduring element of individuals and collectivities. Some of these scholars have emphasized the linkages of identity to social structures, whereas others have mostly focused on individual cognitive processes [82]. The seminal work by Albert and Whetten [3] exemplifies this tradition in organization studies. Some in the field of medical education [27–30] have espoused this earlier view of social identity theory. For these authors, professional identity would be the *outcome* of the process of socialization, the *ideal* to be reached by medical trainees aspiring to belong to a *community of practice*. Its establishment would constitute the major learning objective for educators in healthcare including nursing [78] and other health professions.

Yet other scholars, from a variety of disciplines, have attributed a different ontological meaning to the concept of identity. For them, identity is not a fixed, measurable entity but a phenomenon socially and historically constructed and constantly subject to contradictions, revisions, and change. According to this view, identity is an ongoing process that encompasses the ‘sense of self’ through social interactions and relates to how social actors understand and explain themselves through dynamic interactional processes [40, 42, 62, 87]. The accomplishment of these interactional processes would be mainly made through language-in-use; professional discourse is therefore fundamental to the construction of their own identity and of the space and role they occupy in the social world [68]. Rodgers and Scott [71] have clearly summarized this later view of identity as follows: “Contemporary conceptions of identity share four basic assumptions: (1) that identity is dependent upon and formed within multiple *contexts* which bring social, cultural, political, and historical forces to bear upon that formation; (2) that identity is formed in *relationship* with others and involves *emotions*; (3) that identity is *shifting, unstable, and multiple*; (4) that identity involves the construction and reconstruction of meaning through *stories* over time” (p. 733).

*Levels of representation.* When accepting that identity is constructed through social interactions, two other levels of representation of the self besides the *individual* level must be considered, i.e., *interpersonal* and *group* levels [13], social identities being constructed through both interpersonal relationships and collective identities. This is the case for the healthcare professional identity, which can be constructed and reconstructed through the doctor-patient, doctor-nurse, or doctor-‘other health provider’ relationship, but also in terms of membership in the social category of a particular health care profession [16].

A profession is an occupation characterized by both the possession of a specialized body of knowledge and a commitment to service [75, 83]. Any health profession “reflects internalizations of the norms and characteristics of important reference groups and consists of cognitions about the self that are consistent with that group identification” [13], p. 84. This process of differentiation, which once again comes into being through language-in-use, implies attaching value to a specific group membership [6] and simultaneously a separation from other social groups [2]. A professional identity is therefore created and recreated through professional discourse constituted by everything professionals do in the day-to-day accomplishment of their responsibilities and tasks [75]. Such a discourse would be “not only durable, but also legitimate and authoritative” (p. 15).

Professional discourses do not emerge in a vacuum. On the contrary, the fleshing out of professional identity through discursive activity can only be understood and explained within the *context* in which social interactions occur. In this regard, we agree with Sarangi and Roberts [75] when they point out: “What counts as legitimate professional discourse will depend on the range of discourses available within an institutional order” (p. 15). Context is thus crucial for both understanding discourses and creating identity [42].

*Gender issues in professional identity formation.* Gender is a social dimension fully embedded in processes of professional identity formation and constant development. The health professions here at stake are, in fact, quantitatively feminized: nursing has always been a predominantly female profession, and women are increasingly represented in the family physician workforce. As noted by the Canadian Institute for Health Information (CIHI) in its *Physicians in Canada, 2019 report*: “In [19], 47.5% of family medicine physicians and 38.0% of specialist physicians in Canada were female. Since 2015, the number of female physicians in the workforce has increased by 19.2%, while the number of male physicians has increased by 5.8%.” According to the same report, “Quebec had the highest proportion of female physicians, at 50.6%,” These statistics were corroborated by data from the Canadian Medical Association [17] that nation wide, 53.4% of family physicians were male and 46.6% female. Moreover, two thirds (64%) of family physicians under age 35 were female. This feminization trend in the medical profession has been further confirmed by the latest CIHI report [20], in which is it stressed that: “In [17], 49.7% of family physicians and 40.2% of specialist physicians in Canada were female.”

Despite this portrait, evidence still points to gender inequities, even in health professions that are feminized such as nursing. For instance, Greene et al.’s [36] investigation on USA nurse practitioner salaries demonstrated that, whereas female nurse practitioners are overwhelming more numerous than male counterparts (similarly in Canada), the latter earn “significantly more than female NPs across all clinical specialty areas.” Moreover, a recent scoping review on professional identity formation (PIF) conducted by Volpe et al. [86] revealed that there is a lack of empirical work on PIF in which “trainee’s sociocultural data, such as race, ethnicity, gender, sexual orientation, age and socioeconomic status” are considered in a “robust way”. In a similar vein, studies on professional identity construction in primary care work settings are not only scarce but also lack robust gender analysis.

#### Family physicians

How FPs see themselves as medical professionals, and how they behave accordingly in their interactions with patients and other health professionals has significantly changed in the 21st century. Prevailing trends influencing clinical practice include epidemiological shifts, the increasing role of information technology and artificial intelligence, the feminization of the profession, the increasing specialization of the medical profession, the need for interprofessional co-operation and coordination for optimal care delivery, are perceived *identity threats* to the more traditional solo practice in which the physician is at the top of the primary health care hierarchy. The topic of PIF in academic centers has also attracted much attention in the health sciences education field [25, 61]. However, as noted by Volpe et al. [86], “the empiric study of PIF in medicine remains in its infancy”, scholars having mainly focused on medical trainees, either students [48, 72] or residents [69, 76]. There is a dearth of empirical work on the way that practicing family physicians have faced identity threats and have reconstructed their professional identity in organizations, institutions, and societies in constant motion. An exception is the work of Kyratsis et al. [53], who examined the identity threats experienced by family physicians from five European countries transitioning from a former Soviet mode of specialized medical practice to a more generalist Western practice of family medicine.

#### Nurse practitioners

NPs’ professional identity construction is a complex process that first implies the formation of the professional as an ‘expert nurse’ to then transition to an ‘NP’, which incorporates both nursing values and additional care activities (e.g., diagnosis and prescribing) that were previously fulfilled by physicians [66]. This creates a heightened sense of identity confusion between nursing and medicine that persists well into the first year of practice following graduation [34]. Chulach and Gagnon [24] characterized the NP role as hybrid.

NPs were introduced in Canada in the 1960s [46]. There are currently 6 661 NPs in Canada [18]. Ontario is the province in Canada with the largest number of NPs with 3451 [18] while there are currently 842 NPs in Québec [65]. NPs in training and in the first years of practice have consistently reported that integrating portions of the medical identity associated with their role (hypothetic-deductive reasoning and diagnosis) is challenging and takes time [49, 50]. In rural areas, Owens [66] found that NPs experienced an increased sense of responsibility to provide access to care for patients and communities. Internationally, challenges experienced by physicians, NPs, inter-professional team members and decision-makers when adding new NP roles to inter-professional teams are well documented [44, 45, 50]. Roles where NPs are used to their full potential lead to better outcomes for patients (e.g., blood pressure control) [50, 64]. However, physician understanding and acceptance of NP roles and the activities they share is crucial to developing role clarity and role identity [50].

## Methods/design

### Theoretical framework

This study will be theoretically nourished by social identity theory as reinterpreted in organizational studies. This perspective will allow us to pay attention to the three levels of representation that comes into play in professional identity formation, namely *individual* (FP, NP), *interpersonal* (in this study, the focus will be on the dyadic interactions between FPs and NPs), and *group* (FPs’ and NPs’ double sense of belonging to the family medicine and nursing professional groups. and interdisciplinary primary care team). This theoretical approach has informed not only the conceptualization of the study, but also the development of the research plan detailed below, and then its execution.

**Fig. 1:**
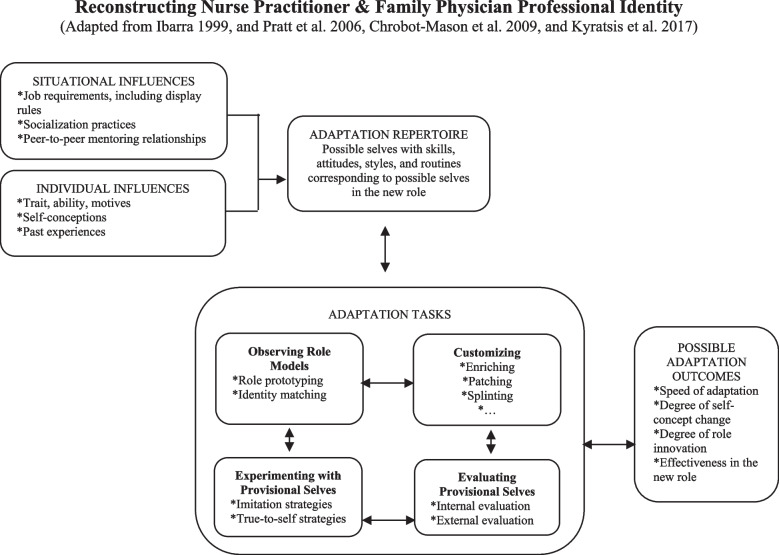
Reconstructing Nurse Practitioner & Family Physician Professional Identity [23, 43, 53, 69]

We fully adhere to the definition of identity stated by Rodgers and Scott [71] and adopt *the process theory of provisional selves* proposed by Ibarra [43], enriched with the triggers of social identity conflicts as suggested by Chrobot-Mason et al. [23], and the adaptation strategies proposed by Pratt et al. [69], Chrobot-Mason et al. [23]; and Kyratsis et al. [53] – see also Fig. 1. Under a variety of individual and situational influences, and in the presence of a number of triggers seen as identity threats, evolving ‘image’ and ‘identity’ repertoires (discourses) existing in work contexts will have an effect on the choices available to NPs and FPs to recreate their professional identities as members of an interdisciplinary primary care clinical team. This presumably difficult transition may involve a number of tasks, what Ibarra calls ‘adaptation process’: (1) observing role models to identify potential identities to emulate; (2) customizing how they see themselves and how they have to behave; (3) experimenting with provisional selves; (4) evaluating experiments against internal and external feedback. A set of possible adaptation outcomes could then emerge in terms of identity reconstruction, both in terms of self-concept change and ways to behave.

Alvesson and Willmott [4] have also emphasized the role of discourse in processes of identity construction and reconstruction in their model of identity regulation as organizational control. For these recognized critical management scholars, organizational discursive practices contribute to developing employees’ self-images and work orientations coherent with managerial objectives in order to pursue organizational control through the regulation of identity. Nevertheless, the most critical issue is the *linking* of discourse to processes of self-identity formation and reproduction. Regulation through the management of identity is conditional upon the strengthening of this link. Yet, to repeat, discourse may be produced and circulated without ‘sticking’ to their targets” [4]; p. 628.

### Research design and description of cases

This is an interpretive, multilevel, and comparative multiple case study [80, 81] (*n* = 2). The main unit of analysis is the *dyadic relationships* between FPs and NPs currently working in primary care teams for at least one year. In congruency with the theoretical approach adopted, two additional levels of analysis are considered, i.e., individual and group. Looking for cases that offer “a good opportunity to learn” [79], members of both primary care clinical teams, located in urban primary care centers, have worked together for more than a decade. To maximize transferability whilst balancing feasibility, cases come from two different Canadian provinces, i.e., Quebec and Ontario. This choice allows working in different primary care organizations – the organizational setting in which primary care teams operate might have an effect on the way teamwork will develop, so it is plausible to consider that FPs’ and NPs adaptation processes will also be diverse. All these features will allow comparisons and help generate “robust and reliable results” [9, 39]. Further, to attain a rich understanding of the processes at stake, i.e., processes of FPs’ and NPs’ professional identity reconstruction, we adopt a longitudinal design, our fieldwork covering two consecutive years from 2024 to 2026.

### Participants, sampling strategies, and recruitment

According to a purposeful sampling approach, and in congruency with the focus of this research project, key informants in this study are FPs and NPs currently working in the two primary care teams involved in the study. There usually are two or three NPs in each one of the sites, who are all selected for the study, as well as at least the two or three FPs with whom they have more frequent contact, i.e., about *n* = 12, 6 per site. Based on prior research on NPs in Quebec [49, 50], we have estimated that we will examine about 8–10 dyads across the two sites, i.e., about 4–5 dyads per site.

Furthermore, three other groups of primary care stakeholders will be invited to also participate in the investigation: the Directors of the two primary care teams involved in the study, other clinicians of the primary care team, and adult patients. We follow a stepwise recruitment strategy to recruit all the participants in the investigation, starting with the site director of the primary care clinic, then the NPs and FPs, then patients rostered to participating NPs and FPs, and finally members of the primary health team (MDs, RNs) who are not key informants in this study. With the formal agreement of the Directors of the respective primary care teams, we have organized an information session about the purpose of the study as well as fieldwork requirements of the investigation. We have also reassured potential participants about confidentially and anonymity of data.

Then, at the end of each year of the research period considered, we will select 6–10 rostered patients and additional 6–10 clinicians for focus groups. To do so, we will engage in maximum variation sampling according to the following criteria: (1) age, (2) gender, (3) time enrolled in the clinic.

### Data collection

The first qualitative method that we will use for data generation will be individual semi-structured interviews with the Directors of the primary care teams involved in the study (*n* = 2) [52]. These ‘conversations with purpose’ with the primary care team managers will pave the way to the subsequent phases of the fieldwork. Second, in congruency with the longitudinal design of this investigation, the most original technique for data generation will be the recording of an audio-diary [26, 59] by the NPs and at two-three FPs working in the same team. These primary care professionals will be asked to record their exchanges with the rest of the members of the interdisciplinary team, in particular their dyadic relationship, and their reflections about the influence of their experiences and meanings on themselves as NPs or FPs over the whole two-year research period. They will be requested to do so using the Otter.AI device. Although its use is still infrequent, solicited audio diary is nonetheless “a powerful tool for researchers interested in narrative enquiry. It opens new insights into the way in which we make sense of the world of telling our stories to another and ourselves” [60]; p. 89. Third, the professionals involved in the investigation will also be met by the researchers at the end of each year over the two consecutive years considered for an in-depth individual qualitative interview [55]. Inspired by the theoretical framework adopted, the annual conversations with these participants will allow us to delve into and bring to light their ideas about interdisciplinary primary care practice, their expectations and the meaning they give to their dyadic NP-FP relationships, and their respective adaptation processes: how their view about themselves changes over time, the evolving meaning they give to be “a family physician” or “a nurse practitioner”, and possible events or processes that have triggered change, their ideas about their professional roles, and the arguments they provide to explain their career path and how they evolved during the research period. The total number of interviews to conduct with these professionals will thus be about 4–5 interviews/year/2 sites/2 years = 16–20. The completion of a prior demographic questionnaire by all interviewees will help better understand these professionals’ individual influences on their respective identity work. Fourth, we also plan to facilitate two focus groups [51] per year: one with members of the whole primary care team, and another one with patients. The focus group technique allows data to emerge from the interaction among participants and have demonstrated to be more effective than individual interviews in personal and sensitive disclosures [38]. Consequently, focus groups fit well with the co-constructed and performative nature of identity. Focus groups usually comprises 6–10 participants that discuss and exchange ideas about a topic of common interest. We plan to conduct *n* = 2 focus groups/year/site = 8. Participants will be selected according to a maximum variation sampling strategy: in terms of age, sex/gender, health discipline, years of practice, and cultural and academic background for focus groups with primary care providers; in terms of age, sex/gender, acute/chronic health condition, and cultural background for focus groups with patients. Focus groups will be conducted in the fall of 2024 and 2025 and moderated by a researcher, assisted by the research trainees. To ensure validity of the data collected, debriefing meetings among the members of the research team will take place immediately after each focus group. Fifth, we also plan to use non-participant observations to supplement data gathered through auto-diary records and interviewing [35]. We will take advantage of scheduled visits to conduct focus groups to spend 1 week/year on average in each one of the FMGs involved in the study and observe physician-nurse interactions during, for instance routine clinical work, administrative encounters, and continuing professional development events. Finally, organizational documents will constitute another important source of data because they “provide background and context, additional questions to be asked, supplementary data, a means of tracking change and development, and verification of findings from other data sources [12]. There are many different types of documents that will be significant for our investigation: minutes of clinical and administrative meetings, other administrative publications, newspapers and magazines, charts, tables, lists, etc. Documentary sources will help elicit nuanced meanings and interpretations [41, 67] and will broaden the understanding of the team and primary care organization contexts that surround the professional identity adaptation processes.

### Data analysis

In congruence with the nature of the phenomena we will examine, the theoretical approach adopted, and the methodology chosen, we will adopt a broad array of *discursive techniques* for qualitative data analysis as data is longitudinally gathered. To answer the first research question (i.e., what is the meaning that FPs and NPs give to their respective professional identities in primary care? ), we will apply a *narrative analysis* to auto-diary records and verbatim transcriptions from individual interviews and focus groups interviews with physicians and nurses. We will put emphasis on what is said [70], as well as on the temporal organization of the narrative [54]. To answer the second research question (i.e., what and how do individual, team, organizational, and institutional factors influence their FPs and NPs identity work in interdisciplinary primary care teams? ), we will apply a *performative narrative analysis* of diary notes, diary records, and verbatim transcription from individual interviews and focus groups because our interest here goes “beyond the spoken word”; as noted by Riessman [70]: “The performative view is appropriate for studies of communication practices, and for detailed studies of identity construction – how narrators want to be known, and precisely how they involve the audience in “doing” their identities” (p. 5). In all this material we will combine narrative analysis and *systematic metaphor analysis* [77]. Metaphors are figures of speech through which something is named as if it was something else [56, 57]. We propose to perform this analysis because metaphors constitute very powerful discursive practices through which “future identities are made” [31]. While privileging a rather inductive initial coding phase that is consistent with Stakes’s case study methodology, the theoretical framework described above will sustain the soundness of subsequent interpretations and explanations of results. Documents, in turn, will be analyzed following an iterative process into three phases, namely skimming (superficial examination), reading (thorough examination), and interpretation [12]. The development of interim within- and cross-case research reports through the triangulation of findings [63] will be elaborated after the first year of fieldwork, and final within- and cross-case reports will be written at the end of the study.

### Strategies for assuring trustworthiness

Familiarization with the contexts in which fieldwork will be conducted, regular team debriefing, triangulation of methods, and member checks will be the privileged means for guarantying credibility. Careful purposeful selection of participants, thick descriptions of both the corpus of data generated and the different contexts in which data was generated, and the adoption of an established theoretical framework appropriate to the phenomenon at stake are the strategies of election to assure transferability. The establishment of an audit trail per case will sustain dependability. Confirmability will be strengthened via triangulation of methods and the practice of reflexivity throughout the whole research process [37].

## Discussion

We aim to conduct the proposed investigation with the ambition to generate new significant knowledge for both practice and theory in health services research and health professions education. As detailed in the description of the project, identity is a very important topic in both organization studies and health professions education. The findings of this study will theoretically enrich both bodies of knowledge by focusing on the examination and understanding of processes whereby autonomous primary care providers reconstruct their respective professional identities when accomplishing similar tasks in healthcare delivery.

For practice, the results of the investigation will:

- be highly relevant for FPs, NPs, managers, and patients of primary care multidisciplinary teams to better understand and sustain identity issues, which ultimately will improve the global performance (health outcomes) of their organization;
- be instrumental for the directors of postgraduate training programs in family medicine, nursing, and interdisciplinary health sciences to constantly improve their educational interventions regarding professional identity formation;
- be translated into postgraduate and continuing professional development initiatives to support professional identity adaptations processes, not only for FPs and NPs but also other primary care providers with whom these professionals interact, e.g., pharmacists.
- through their diffusion and dissemination, inspire the evidence-based implementation of similar initiatives in primary care organizations elsewhere in Canada, and academic contexts at the international level.

## Data Availability

No datasets were generated or analysed during the current study.

## Abbreviations

FPs: Family physicians
NPs: Nurse practitioners
PIF: Professional identity formation
